# Supplementary material for: Molecular basis of specificity and deamidation of eIF4A by Burkholderia Lethal Factor 1
Source: Commun Biol. 2022 Mar 28;5:272. doi: 10.1038/s42003-022-03186-2 (PMC8960835; doi:10.1038/s42003-022-03186-2)
Supplement: Supplementary file 2 — Supplementary Information [file 42003_2022_3186_MOESM2_ESM.pdf]

Molecular basis of specificity and deamidation of eIF4A by Burkholderia Lethal Factor 1

Supplementary Information

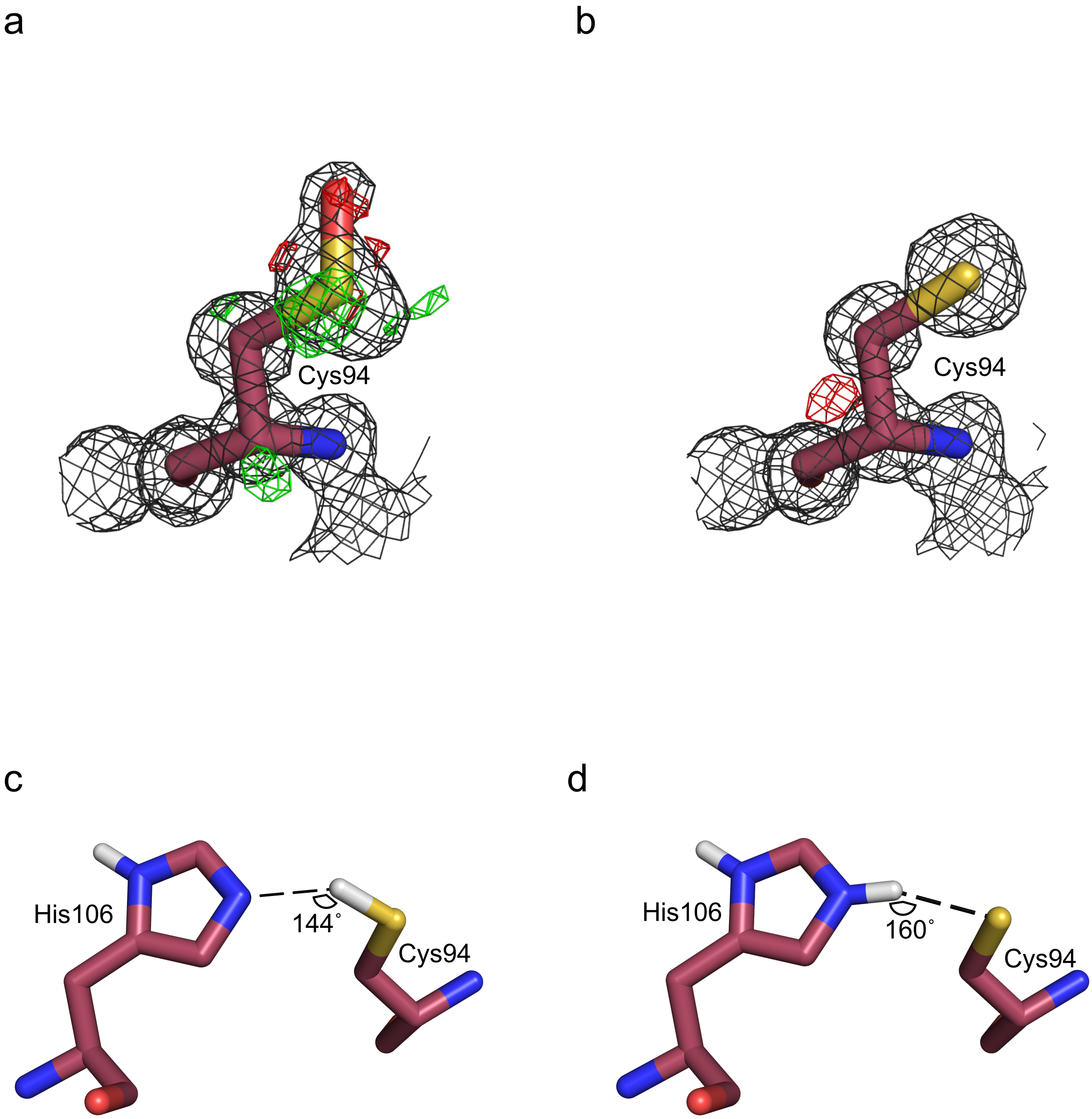

Figure S1

Figure S1: Electron density and hydrogen bonding analysis of BLF1 C94

a) Final 2Fo-Fc and Fo-Fc electron density maps contoured around Cys94 of the WT BLF1 structure (PDB:3TU8) indicating the partial oxidation of the sulphur. b) Equivalent electron density maps surrounding Cys94 of the WT BLF1 structure (PDB:6RVU) showing the absence of oxidation of the side chain. c and d) Schematic diagrams to highlight the deviation from linearity of the hydrogen bond between the side chains of Cys94 and His106 which depends on whether the hydrogen bond donor is Sγ of Cys94 (144˚) (panel c) or Nε2 of His106 (160˚) (panel d). Potential hydrogen bonds are represented as black dashes. 2Fo-Fc electron density maps (grey) are contoured at 1.0 σ, while the Fo-Fc electron density maps (green/red) are contoured at 3.0 σ.

a

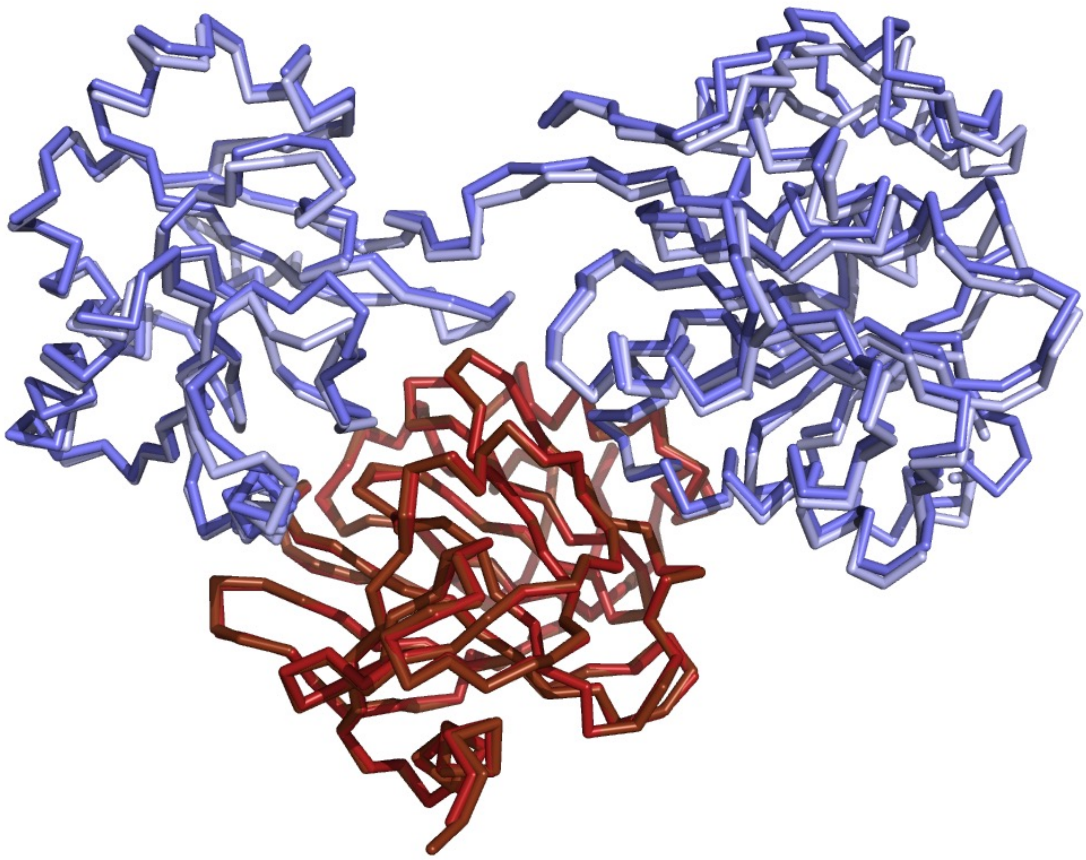

b

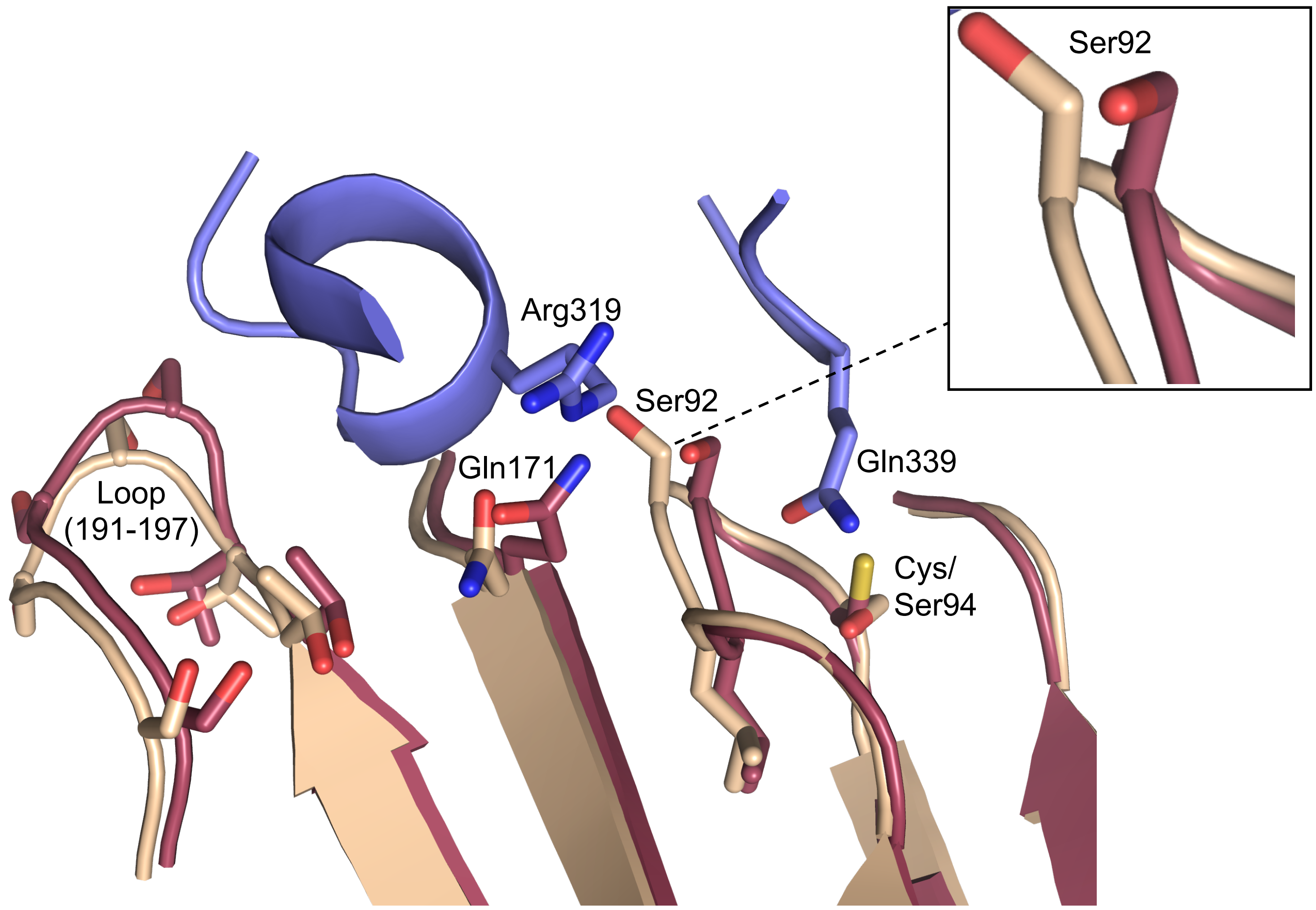

Figure S2

Figure S2: BLF1C94S undergoes minimal rearrangement upon binding eIF4A$^{\Delta20}$

a) Structural comparison of the BLF1 C94S:eIF4A$^{\Delta20}$ complex forms A (PDB:7PPZ (BLF1; maroon, eIF4A; blue)) and B (PDB:7PQ0 (BLF1; chocolate, eIF4A; light blue)) showing the small difference in the position of the C-terminal domains of eIF4A. b) Superposition of the coordinates of BLF1 C94S:eIF4A$^{\Delta20}$ in the form B complex (PDB:7PQ0 (BLF1; maroon, eIF4A; blue)) with those of the WT form of BLF1 (PDB:6RVU; wheat) showing their very similar conformation, but with small shifts in β7-β8 (residues Leu91-Cys/Ser94), Gln171, and the β13-β14 loop (residues Thr191-Ser197). The binding of Gln339 alters the torsion angle of Ser92, leading to other small conformational changes.

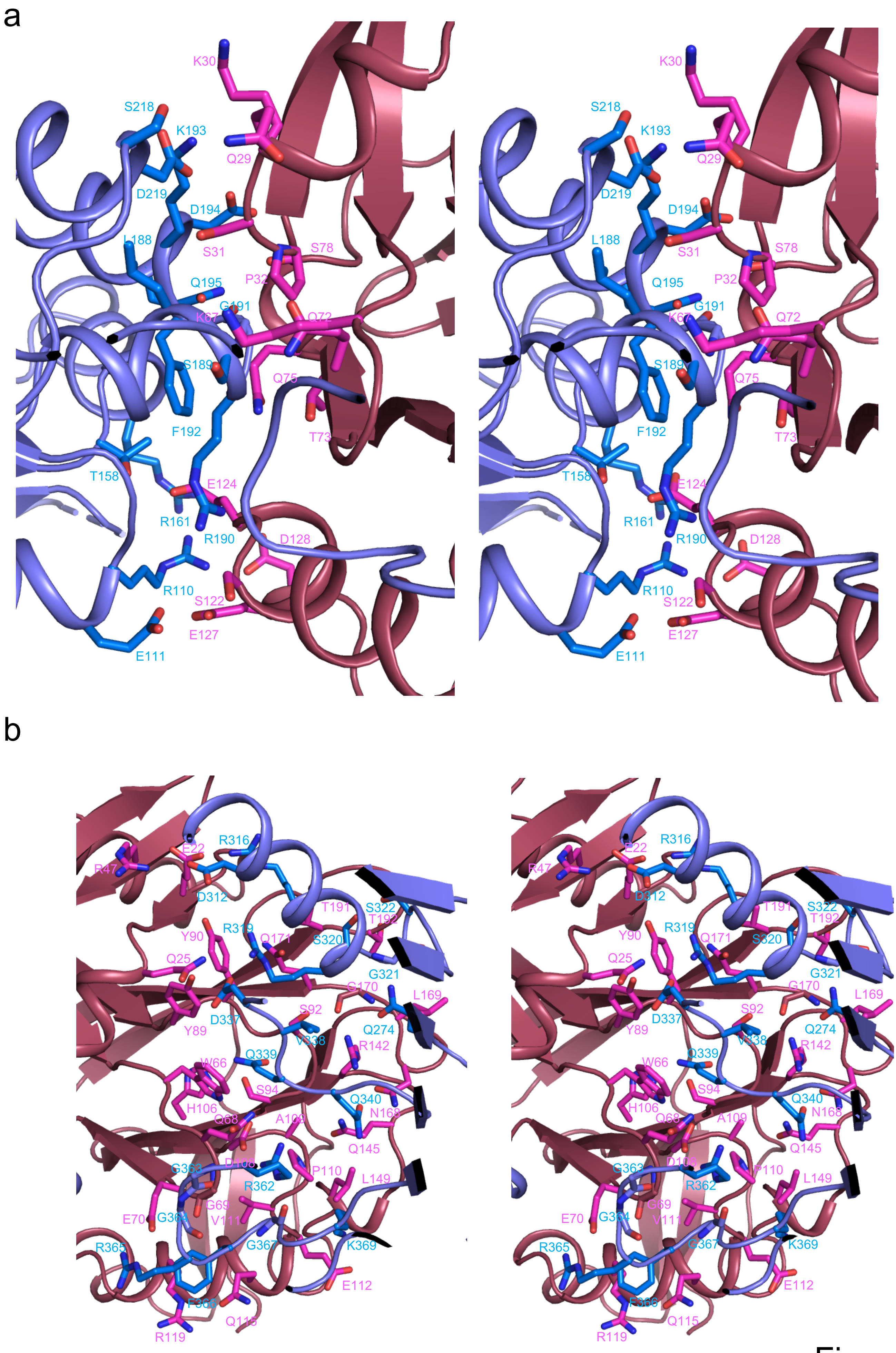
a
K30
S218
K193
Q29
D219
D194
L188
S31
S78
Q195
P32
G191
K67
Q72
S189
Q75
F192
T73
T158
E124
R161
R190
D128
R110
S122
E127
E111
b
R316
E22
R47
D312
T191
S322
T192
R319
Y90
Q171
S320
Q25
G321
G170
L169
D337
S92
Y89
Q274
V338
R142
W66
Q339
S94
H106
Q340
N168
Q68
Q145
G363
P110
L149
R362
G69
E70
V111
G367
K369
R365
E112
R119

Figure S3

Figure S3: Cartoon and sticks stereo-diagrams to illustrate the specificity of BLF1 towards eIF4A$^{\Delta 20}$ showing the network of polar interactions at the interface
a and b) The interacting surface between BLF1 C94S and the N- and C-terminal domains of eIF4A, respectively. Colour scheme: the BLF1 and eIF4A main chain backbones are shown as cartoons in maroon and blue, respectively. Selected side chains are labelled and shown in stick format and coloured by atom (BLF1 C94S; magenta, eIF4A; marine).

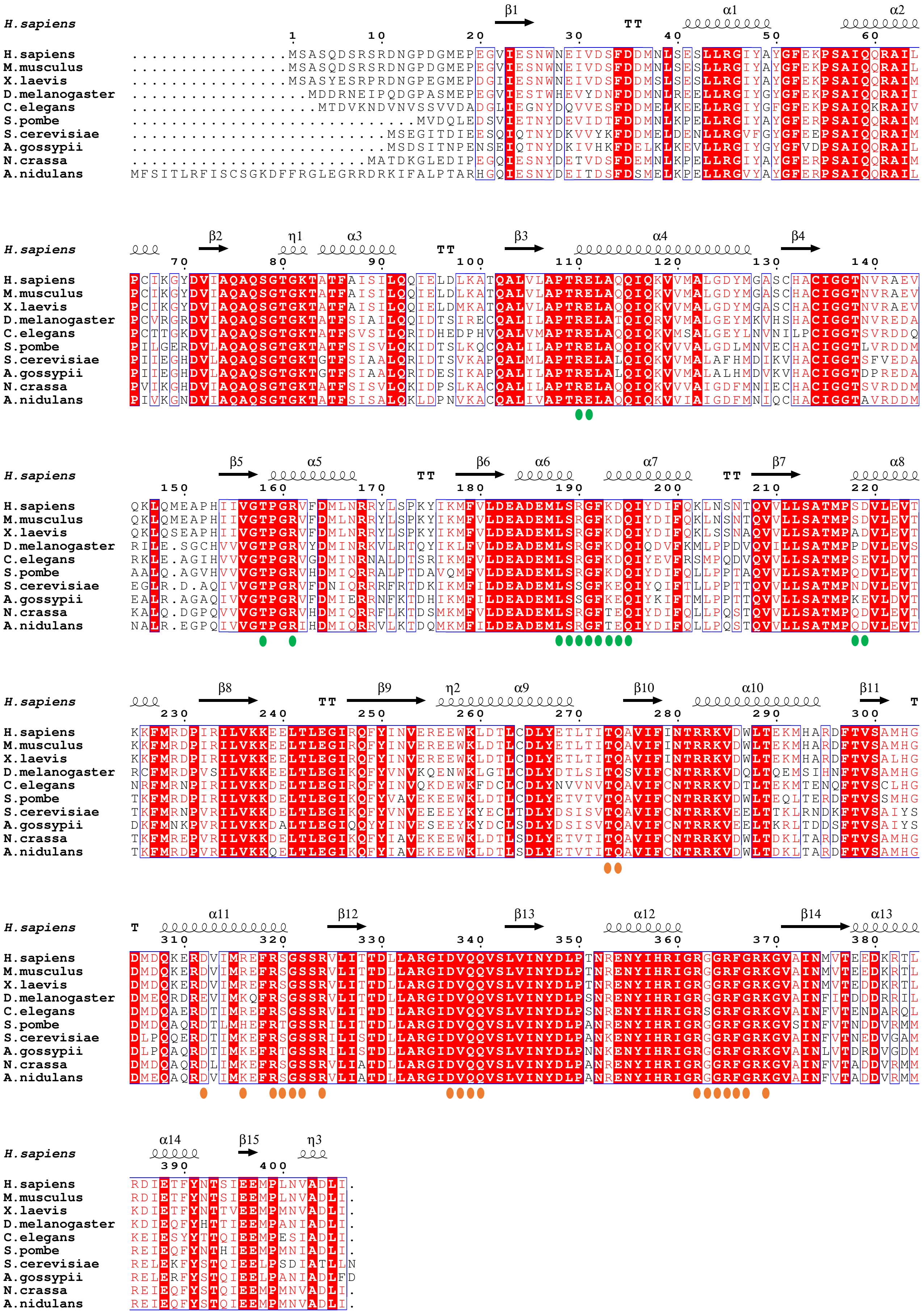
H.sapiens
M.musculus
X.laevis
D.melanogaster
C.elegans
S.pombe
S.cerevisiae
A.gossypii
N.crassa
A.nidulans
β1 β2 β3 β4 β5 β6 β7 β8 β9 β10 β11 β12 β13 β14 β15
α1 α2 α3 α4 α5 α6 α7 α8 α9 α10 α11 α12 α13 α14
η1 η2 η3

Figure S4

Figure S4: A multi-sequence alignment of eIF4A across a representative range of eukaryotes

A cartoon representation of the secondary structures seen in the X-ray structure of human eIF4A in complex with BLF1 (PDB:7PQ0) is presented against a multi-species sequence alignment of eIF4 from fungi to man showing the extensive sequence conservation. Exposed residues on the N- and C-terminal domains of eIF4A whose surface areas change by more than 20 $Å^2$ on formation of the complex with BLF1 are highlighted in green and orange dots below the sequences, respectively. Figure prepared using ESPript with the default colour scheme.

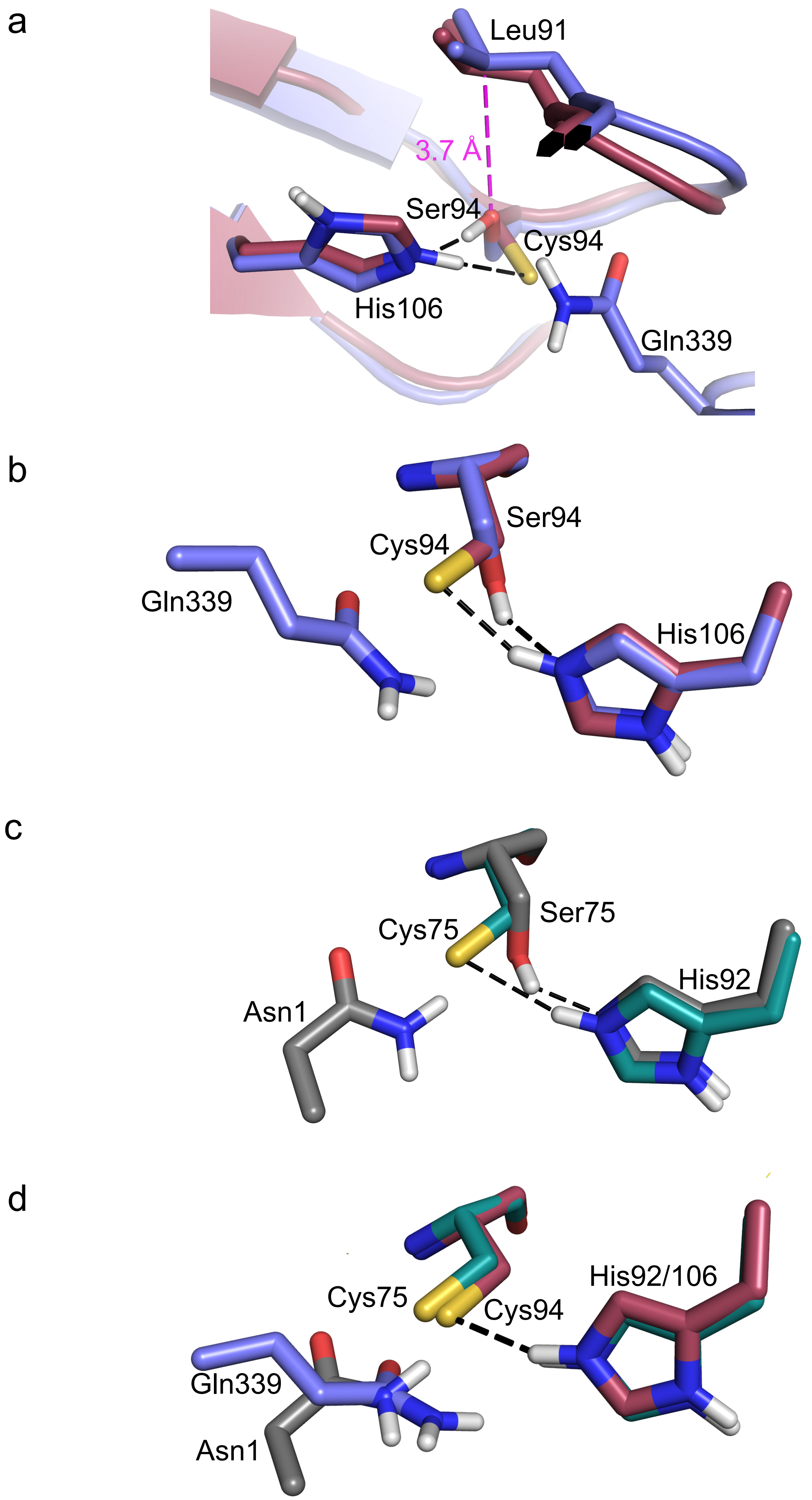
a
Leu91
3.7 Å
Ser94
Cys94
His106
Gln339
b
Ser94
Cys94
Gln339
His106
c
Ser75
Cys75
His92
Asn1
d
His92/106
Cys75
Cys94
Gln339
Asn1

Figure S5

Figure S5: Superposition of the active sites of BLF1 and NTAN1

a) Superposition of the WT BLF1 (PDB:6RVU; maroon) with BLF1:eIF4A$^{\Delta 20}$ C94S mutant (PDB:7PQ0; blue) to highlight the different pattern of hydrogen bonding between $N_{\varepsilon 2}$ of His106 and Cys/Ser94. A steric clash between S_{γ} of Cys94 and C_{γ} of Leu91 would occur if the cysteine adopted the same conformation as the serine (magenta dashes). b) Superposition of the WT BLF1 (PDB:6RVU; maroon) with the BLF1 C94S:eIF4A$^{\Delta 20}$ complex (PDB:7PQ0; blue) to show the relative orientations of the active site cysteine and histidine residues with respect to the substrate amide, highlighting the change in torsion angle that accompanies the cysteine to serine mutation. c) Equivalent superposition of WT NTAN1 (PDB:6A0E; teal) with the NTAN1 C75S:peptide complex (PDB:6A0H; grey) showing a similar pattern of torsion angle changes to that seen in BLF1. d) Local superposition of the active sites in the hybrid models of WT BLF1:eIF4A$^{\Delta 20}$ and WT NTAN1:peptide.

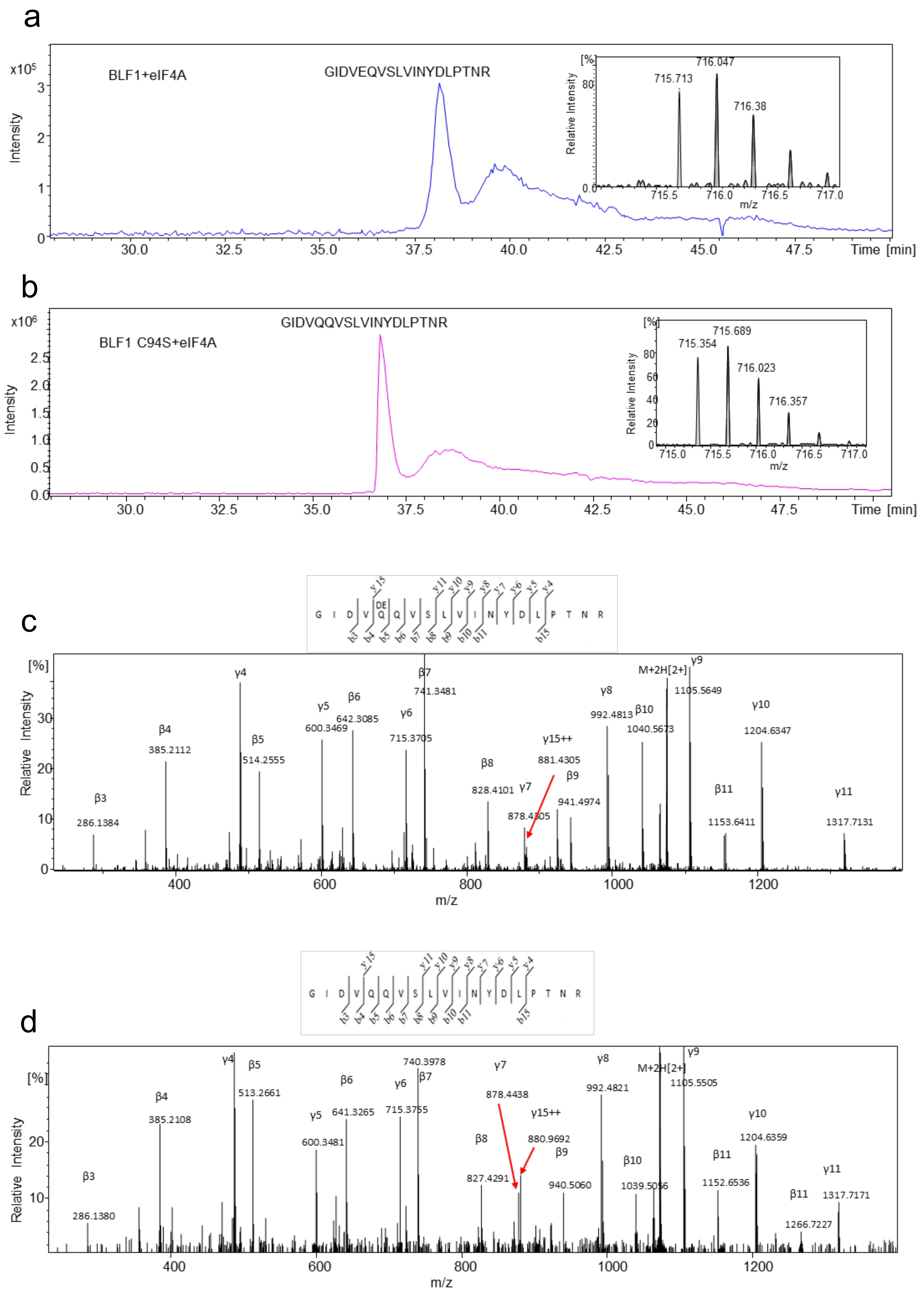
a
BLF1+eIF4A
GIDVEQVSLVINYDLPTNR
Intensity
Time [min]
Relative Intensity
m/z
b
BLF1 C94S+eIF4A
GIDVQQVSLVINYDLPTNR
Intensity
Time [min]
Relative Intensity
m/z
c
Relative Intensity
m/z
M+2H[2+]
d
Relative Intensity
m/z
M+2H[2+]

Figure S6

Figure S6: LC MS analysis of the Gln339 containing tryptic peptide from eIF4A$^{\Delta 20}$

a) Extracted ion chromatogram of the tryptic peptide GIDVEQVSLVINYDLPTNR $[M+3H]^{3+}$ from BLF1+eIF4A. b) Extracted ion chromatogram of the peptide GIDVQQVSLVINYDLPTNR $[M+3H]^{3+}$ from BLF1 C94S+eIF4A, no significant deamidation of eIF4A was observed. MS1 spectra are shown inset. c) MS/MS analysis of the deamidated peptide GIDVEQVSLVINYDLPTNR $[M+3H]^{3+}$ from BLF1+eIF4A. d) MS/MS analysis of GIDVQQVSLVINYDLPTNR $[M+3H]^{3+}$ from BLF1 C94S+eIF4A. The prominent β and γ ions are highlighted confirming the deamidated and non-deamidated peptides respectively.

Table S1: Contacts and surface area changes between BLF1 C94S and eIF4A$^{\Delta 20}$

a) Residues whose surface area changes upon formation of the BLF1:eIF4A complex. Surface areas are rounded to the nearest 10 Å^2. Residues in bold italic text are strongly conserved in a multiple sequence alignment of eIF4A (Figure S4). b & c) Hydrogens bonds (b) and salt bridge (c) formed in the BLF1:eIF4A complex.

a

eIF4A N-terminal domain		eIF4A C-terminal domain	
Residue Number	Surface Area Change (Å^2)	Residue Number	Surface Area Change (Å^2)
Arg110	-90	***Thr273***	-50
Glu111	-30	***Gln274***	-20
Thr158	-20	Asp312	-30
Arg161	-30	Arg316	-50
Leu188	-20	***Arg319***	-110
Ser189	-70	Ser320	-80
Arg190	-90	***Gly321***	-20
Gly191	-50	***Ser322***	-30
Phe192	-30	***Arg324***	-20
Lys193	-50	***Asp337***	-80
Asp194	-70	***Val338***	-30
Gln195	-40	***Gln339***	-170
Ser218	-40	***Gln340***	-120
Asp219	-50	***Arg362***	-60
Total	-680	Gly363	-30
		Gly364	-30
		Arg365	-30
		Phe366	-130
		Gly367	-30
		Lys369	-70
		Total	-1190

b

eIF4A	Distance (Å)	BLF1 C94S
ARG 110 [NH1]	3.2	ASP 128 [OD1]
ARG 110 [NH1]	3.4	GLU 124 [O]
ARG 161 [NH1]	2.8	GLU 124 [OE2]
SER 189 [O]	2.9	THR 73 [N]
ARG 190 [O]	2.9	GLN 75 [NE2]
ASP 194 [OD1]	3.0	THR 34 [N]
ASP 194 [OD2]	2.8	SER 78 [OG]
ASP 194 [OD2]	2.7	THR 34 [OG1]
GLN 195 [NE2]	3.4	SER 78 [OG]
ASP 219 [OD2]	3.1	SER 31 [N]
ARG 316 [NH1]	2.4	GLU 22 [OE2]
ARG 319 [NE]	3.0	GLN 171 [OE1]
ARG 319 [O]	3.7	GLN 171 [N]
ARG 319 [O]	3.7	SER 92 [OG]
SER 322 [OG]	3.8	THR 192 [O]
GLN 339 [NE2]	2.8	TYR 90 [O]
GLN 339 [OE1]	3.1	SER 94 [N]
GLN 339 [OE1]	2.6	SER 92 [N]
GLN 340 [N]	3.7	SER 92 [O]
GLN 340 [OE1]	3.0	GLN 145 [NE2]
GLN 340 [OE1]	3.1	ARG 142 [NH1]
GLN 340 [OE1]	2.9	ASN 168 [ND2]
ARG 362 [NH2]	2.8	ASP 108 [OD2]
GLY 363 [N]	3.1	GLN 68 [OE1]
ARG 365 [N]	3.4	GLY 69 [O]
ARG 365 [N]	3.2	GLU 70 [OE2]
ARG 365 [NE]	2.8	GLU 70 [OE2]
ARG 365 [NH2]	3.2	GLU 70 [OE1]
PHE 366 [N]	2.8	GLY 69 [O]

c

eIF4A	Distance (Å)	BLF1 C94S
ARG 110 [NH1]	3.2	ASP 128 [OD1]
ARG 110 [NH1]	3.9	GLU 127 [OE2]
ARG 110 [NH2]	3.8	ASP 128 [OD1]
ARG 110 [NH2]	3.4	GLU 124 [OE1]
ARG 110 [NH2]	3.9	GLU 124 [OE2]
ARG 161 [NE]	3.9	GLU 124 [OE2]
ARG 161 [NH1]	2.8	GLU 124 [OE2]
ASP 312 [OD1]	3.9	ARG 47 [NH1]
ARG 316 [NH1]	2.4	GLU 22 [OE2]
ARG 362 [NH2]	2.8	ASP 108 [OD2]
ARG 365 [NE]	4.0	GLU 70 [OE1]
ARG 365 [NE]	2.8	GLU 70 [OE2]
ARG 365 [NH2]	3.2	GLU 70 [OE1]
ARG 365 [NH2]	3.5	GLU 70 [OE2]
